# Role of Persistent Organic Pollutants in Breast Cancer Progression and Identification of Estrogen Receptor Alpha Inhibitors Using In-Silico Mining and Drug-Drug Interaction Network Approaches

**DOI:** 10.3390/biology10070681

**Published:** 2021-07-19

**Authors:** Bibi Zainab, Zainab Ayaz, Umer Rashid, Dunia A. Al Farraj, Roua M. Alkufeidy, Fatmah S. AlQahtany, Reem M. Aljowaie, Arshad Mehmood Abbasi

**Affiliations:** 1 Department of Environmental Sciences, Abbottabad Campus, COMSATS University Islamabad, Abbottabad 22060, Pakistan; abbasizainab2@gmail.com (B.Z.); zainabayaz321@gmail.com (Z.A.); 2 Department of Chemistry, Abbottabad Campus, COMSATS University Islamabad, Abbottabad 22060, Pakistan; umerrashid@cuiatd.edu.pk; 3 Department of Botany and Microbiology, College of Sciences, King Saud University, P.O. Box 22452, Riyadh 11495, Saudi Arabia; dfarraj@ksu.edu.sa (D.A.A.F.); ralqufaidi@ksu.edu.sa (R.M.A.); raljowaie@ksu.edu.sa (R.M.A.); 4 Department of Pathology, College of Medicine, King Saud University, Medical City, Riyadh 11495, Saudi Arabia; fatma@ksu.edu.sa; 5 University of Gastronomic Sciences, 12042 Pollenzo, Italy

**Keywords:** breast cancer, estrogen receptor alpha, persistent organic pollutants, drug-drug interaction networks, molecular docking

## Abstract

**Simple Summary:**

The role of persistent organic pollutants (POPs) in breast cancer progression and their bioaccumulation in adipose tissue has been reported. We used a computational approach to study molecular interactions of POPs with breast cancer proteins and identified natural and synthetic compounds to inhibit these interactions. Moreover, for comparative analysis, standard drugs and screened compounds were also docked against estrogen receptor alpha (ERα) and identification of the finest inhibitor was performed using in-silico mining and drug-drug interaction (DDI) network approaches. Based on scoring values, short-chained chlorinated paraffins demonstrated strong interactions with ERα compared to organo-chlorines and PCBs. Synthetic and natural compounds demonstrating strong associations with the active site of the ERα protein could be potential candidates to treat breast cancer specifically caused by POPs and other organic toxins and can be used as an alternative to standard drugs.

**Abstract:**

The strong association between POPs and breast cancer in humans has been suggested in various epidemiological studies. However, the interaction of POPs with the ERα protein of breast cancer, and identification of natural and synthetic compounds to inhibit this interaction, is mysterious yet. Consequently, the present study aimed to explore the interaction between POPs and ERα using the molecular operating environment (MOE) tool and to identify natural and synthetic compounds to inhibit this association through a cluster-based approach. To validate whether our approach could distinguish between active and inactive compounds, a virtual screen (VS) was performed using actives (627 compounds) as positive control and decoys (20,818 compounds) as a negative dataset obtained from DUD-E. Comparatively, short-chain chlorinated paraffins (SCCPs), hexabromocyclododecane (HBCD), and perfluorooctanesulfonyl fluoride (PFOSF) depicted strong interactions with the ERα protein based on the lowest-scoring values of −31.946, −18.916, −17.581 kcal/mol, respectively. Out of 7856 retrieved natural and synthetic compounds, sixty were selected on modularity bases and subsequently docked with ERα. Based on the lowest-scoring values, ZINC08441573, ZINC00664754, ZINC00702695, ZINC00627464, and ZINC08440501 (synthetic compounds), and capsaicin, flavopiridol tectorgenin, and ellagic acid (natural compounds) showed incredible interactions with the active sites of ERα, even more convening and resilient than standard breast cancer drugs Tamoxifen, Arimidex and Letrozole. Our findings confirm the role of POPs in breast cancer progression and suggest that natural and synthetic compounds with high binding affinity could be more efficient and appropriate candidates to treat breast cancer after validation through in vitro and in vivo studies.

## 1. Introduction

POPs are the most common synthetic, lipophilic, toxic, bio-accumulative, and persistent pollutants in the environment. Most POPs are of anthropogenic origin, but some substances, i.e., dioxins and furans, are also produced naturally during volcanism. POPs are also used intentionally in pesticides and other industrial products and may be released accidentally as a by-product from industrial processes or fuel combustion, such as dioxins and furans [1]. POPs release in the environment through industrial and agricultural effluents, drainage systems, urban effluents and landfill leachate [2,3]. Contaminated soil, water, air, dust and processed goods like textiles and packaging materials contain considerable amounts of POPs. Importantly, at ambient temperatures, POPs have a tendency to enter the gas phase; as a result they may volatilize from soils, plants, and water bodies into the atmosphere. They preferentially partition to solids, particularly organic materials in aquatic systems and soils, avoiding the aqueous phase. Being hydrophobic in nature [4], rather than entering the aqueous milieu of cells, some major types of POPs, such as polychlorinated dibenzo-p-dioxins and furans (PCDD/PCFs), polychlorinated biphenyl (PCBs), organo-chlorinated pesticides (OCPs), perfluorooctane sulfonate (PFOS) and pentadecafluorooctanoic acid (PFOAs) are hydrophobic and accumulate in the fatty tissues of the living host. POPs may accumulate in food chains [5] and, from contaminated food such as fruits, vegetables, chicken, meat, milk and fish etc., may enter humans and other living organisms [6,7]. As a result, predatory species like humans often have the highest concentration of POPs, and their presence in humans, i.e., in adipose tissue and human milk, is associated with the up-regulation of hormone-dependent breast cancers [2].

The prevalence of breast cancer, one of the most common types of cancers, specifically in females, is increasing worldwide, which cannot be explained solely by the emergence of mammography screening [8]. In 2018, about two million cases of breast cancer were reported in women globally [9]. The survival rate was up to 26% in cases where distant metastases were present. About 25% of breast cancers have been reported in developed countries; furthermore, it is one of the leading causes of death in Western countries. Deregulation of estrogen balance is known to promote breast cancer, and in Asia, over 60% of breast cancer cases have been diagnosed as estrogen receptor alpha-positive (ERα) cancers [10]. The estrogen receptor 1 (ESR1) gene encodes the ERα protein, a ligand regulated transcription factor, which plays a central role in the proliferation of breast cancer. Production of testosterone enhances the synthesis of progesterone and estrogen receptors in breast glands. Particularly, ERα expressed in the mammary glands and uterus of women has binding ability with DNA and contributes significantly to apoptosis, homeostasis, metabolism, and in breast cancer. An estimated 60% pre- and 75% post-menopausal women are suffering from estrogen-dependent breast cancer [11]. Through disturbing the functioning of adipose tissue, POPs affect the production of estrogens by stimulating genotoxic enzymes and leading to cross-generational epigenetic modifications by modifying the epigenome [12]. Many in vitro studies have shown that certain POPs promote the development of estrogen-positive breast cancer cells by receptor (ER). Exposure to certain POPs, particularly in perinatal studies, can enhance the development of breast cancer and sensitivity to carcinogens and cancerous breast tumors in animal studies. Chemotherapy, hormone therapy, immunotherapy, radiotherapy, and surgery are among the common methods for breast cancer treatment [13], which eventually have multiple side effects. Therefore, it is necessary to find better natural and synthetic compounds for treatment.

In this context, extensive use of anticancer drugs and potential inhibitors with increasing resistance together with numerous side effects highlights an urgent need for novel cancer treatment methods. Therefore, VS methods including negative image-based screening, molecular docking and the pharmacophore hypothesis could be effective tools for identification and screening of the ligands against ER-α receptor. Recent studies have demonstrated that VS methods have the ability to provide structural insights into complex interactions for repositioning and remediation [14], specifically using natural and synthetic compounds [15]. At present, in-silico methods for drug designing, receptor mapping, molecular modeling, and homology modulation etc. are gaining tremendous popularity in drug development, molecular biology, nanotechnology and biochemistry domains. In addition, these methods are used to complement in vitro and in vivo toxicity assessments, particularly to reduce the need for animal monitoring, costs, and time [16]. Furthermore, in-silico cancer modelling opens up new avenues for research into oncogenesis in different biological dimensions and systems. These approaches can assist in expediting the development of diagnostic and therapeutic technologies for clinical care. With reliable digital representations of cancer, the consequences of therapeutic treatments at both the molecular and surgical scales may be anticipated in silico without exposing patients to danger. Previously, an in-silico drug discovery technique exposed that a potential ligand, 1,2,3,4,6-penta-O-galloyl-β-d-glucopyranose, which is a naturally occurring tannin, can inhibit the activity of Ror1 (protein) that contributes significantly to cancer growth and proliferation [17].

Many complementary resources, including microarray, protein-protein interaction, and protein complexes, are being used to discover enriched biological processes and pathways. One example of this is graph theory, which is being used to analyze the lung cancer protein-protein interaction network (PPIN), and to discover highly dense modules which are potential cancer-associated protein complexes [18]. Previously, flavonoids have been proven as potential anticancer agents by virtue of molecular binding to some key targets such as aromatase, fatty acid synthase, xanthine oxidase, cyclooxygenase, lipoxygenase, ornithine decarboxylase, protein tyrosine kinase, phosphoinositide 3-kinase, protein kinase C, topoisomerase II (ATP binding site), ATP binding cassette (ABC) transporter, and phospholipase A2 [19]. The present study was conducted with the aim of determining the molecular interactions between ERα (target) and POPs which were considered as key factors in breast cancer progression. Moreover, for comparative analysis, standard drugs and screened compounds were docked against ERα and the finest inhibitors (natural and synthetic compounds) were identified using in-silico mining and DDI network approaches.

## 2. Materials and Methods

### 2.1. Disease Selection

Breast cancer (BC) was the target disease because of its prevalence around the globe. Currently, more than two million cases of breast cancer have been diagnosed in women [9], while in Pakistan BC is diagnosed in over 90,000 women annually, out of which 40,000 will not survive [20].

### 2.2. Identification of the Mutated Gene

Gene identification was completely disease specific. The GeneCard (www.genecards.org/ (accessed on 20 November 2020)) was used along with a literature review to determine a list of mutated genes involved in breast cancer as reported earlier [21]. Based on GeneCard, the estrogen receptor gene (ERg) was identified as a mutated gene of breast cancer.

### 2.3. Selection and Preparation of Targeted Protein

The Protein Data Bank (PDB), a global database providing the 3-D structure of biological molecules like proteins, DNA, and RNA, was used to select and prepare the targeted protein following the method of Rose et al. [22]. Protein Bank RCSB (https://www.rcsb.org/ (accessed on 21 November 2020)). was used to get the 3-D structure of the ERα (ERα/pdb id: 5W9D) protein of breast cancer (Figure S1). The protein selection was entirely based on mutated genes and the MOE tool was used to prepare the protein file while removing water molecules and attached ligands while hydrogen atoms were added. Afterward, a discovery studio was used to visualize the protein structure.

### 2.4. Validation of Virtual Screening (VS) Protocol

To validate whether our approach can distinguish between active and inactive compounds, we have performed a virtual screen (VS) experiment using actives (627 ERα inhibitors, i.e. binders) as positive control and decoys (20,818 compounds, i.e. non-binders) as a negative dataset obtained from the database of Useful Decoys: Enhanced (DUD-E). All the dataset compounds were docked into the binding site of ERα (PDB ID: 5W9D). The ligand enzyme complexes with the lowest binding energy were analyzed by the MOE ligand interaction module. Finally, Discovery Studio Visualizer was used for the 3-D interaction plot.

### 2.5. Screening and Toxicity Detection of Pollutants

POPs, whose emissions and/or output can be eliminated, or at least reduced substantially, were screened from the list as demonstrated in the Stockholm Convention in 2001. The online server ‘admetSAR’ containing 27 predictive models [23] was used to check the toxicity of screened POPs, and all were lying under toxic classes I, II, and III.

### 2.6. Preparation of Ligand and Molecular Docking

PubChem offers free access to information and biological functions about chemical substances. The database contains chemical information from individual PubChem data providers and the integrated database contains a distinction between chemical structures and the database of substances [24]. PubChem (https://pubchem.ncbi.nlm.nih.gov/ (accessed on 12 June 2021)) was used to extract 3-D structures of screened ligands (POPs) while adopting the previously reported procedure [24]. Afterward, ligands were prepared using the molecular operating environment (MOE) tool.

The molecular docking (MD) technique for the identification and optimization of drug candidates was used to analyze and simulate molecular interactions between the ligand and targeted macromolecules as reported formerly [25]. The MOE tool was used to evaluate the mechanism of molecular interactions between ligands including POPs, approved drugs (positive control), progesterone and testosterone (negative control), and drug candidates (natural and synthetic compounds) with an ERα receptor protein, while Discovery Studio software (DS 4.1) was used to visualize the 3-D interactions following the method as reported earlier [26].

### 2.7. Collection and Mining of Natural and Synthetic Compounds

The ZINC database (zinc.docking.org/ (accessed on 12 July 2021)) was used for the collection of natural and synthetic compounds along with their chemical properties, including Zinc ID, molecular weight, hydrogen bond donors log p, polar dissociation, rotatable bonds, a-polar dissociation, and hydrogen bond acceptors. Lipinski’s rule of five was applied to the collected drug dataset for mining the natural and synthetic compounds as described earlier [15].

### 2.8. Cluster Formation

Weka, a platform for clustering, association, pre-processing, regression, classification, and screening of data [27], was used for clustering of the drug dataset based on a k-means algorithm (k-mean) clustering system. According to the properties of drugs, this method tracks a modest and quick way to categorize a particular record “x_1_, x_2_, x_3_ ... x_*n*_” to numbers of k clusters (k ≤ *n*), where k represents clusters and the row is denoted by *n*.

### 2.9. Drug-Drug Interaction (DDI) Network

Gephi, a primary platform for data analysis and the fastest graphical visualization of large networks [28], was used to create DDI networks from k-means clustering to find a strong connection between drug networks within each cluster. DDI networks were generated based on statistical parameters such as modularity (Ml), path lengths (PL), average degree (AD), average weighted degree (AWD), degree distribution (DD), and graph density (GD). Each network has borders E, vertical V, average path length L and node D, as well as network density and modularity classes. Most strongly-associated natural and synthetic compounds were docked against the targeted ERα protein to identify scoring values/binding energies. Standard breast cancer drugs i.e., Tamoxifen, Arimidex, Letrozole were used as a control to compare the scoring value of screened drugs.

## 3. Results and Discussion

### 3.1. Validation of VS and Reliability of MD

To validate whether our approach can distinguish between active and inactive compounds, a virtual screen (VS) experiment was performed using actives (627 ERα inhibitors i.e. binders) as positive control and decoys (20,818 compounds i.e. non-binders) as negative dataset obtained from the database of Useful Decoys: Enhanced (DUD-E) [29]. All the dataset compounds were docked into the binding site of ERα (PDB ID: 5W9D). Computed binding energy values of the active compound dataset were in the range of 28.6573 to −355.9801 kcal/mol and chemical structures of the most active binders are given in Figure S2. The binding energy values of the decoy set were in the range of −1.0988 to −3.0371 kcal/mol. Therefore, these findings suggest that our VS protocol can distinguish between active and inactive compounds.

The reliability of docking accuracy was assessed in two steps. In the first step, redocking of the native ligand was performed (Table S1). In the second step, a cross-docking experiment was carried out (Table S2). Three-dimensional structures of five estrogen receptor-alpha (PDB accession codes = 1A52, 3ERT, 1GWQ, 1UOM and 5W9D) were retrieved from PDB. In self-docking experiments, all the native ligands were extracted from receptors and root means square deviation (RMSD) was calculated for each re-docked and experimental native ligand [30]. Docking was carried out using the Triangle matcher algorithm (placement stage) and scored by the London dG scoring function [31]. Subsequently, best-scored poses were submitted to a rigid receptor protocol (refinement stage). Throughout the validation of docking protocol, the best performance in terms of computed RMSD value, conformation, binding energy, position, and pose (orientation) was obtained with the Triangle matcher London dG scoring function. The final score was calculated with the ASE scoring function. The whole validation process is presented in Supporting Information (Tables S1 and S2).

### 3.2. Interactions between POPs and ERα

Carcinogenesis is not a simple process; it involves initiation, promotion, and progression [32] of malignancy. The ERα gene is more likely to be involved in cell proliferation and is considered the most popular target to treat breast cancer. As per previous reports, POPs may not directly cause cancer, but act as co-carcinogenic agents [33]. It has been reported that organo-chlorines such as dichlorodiphenyltrichloroethane (DDT), hexachlorocyclohexane (HCH), aldrin, dieldrin, and polychlorinated biphenyls (PCBs) have the potential to stimulate breast cancer cell proliferation through the estrogenic pathway [34]. The association between organochlorine and PCBs exposure and risk of breast cancer has been reported. In the present study, substantial data retrieved from the molecular operating environment (MOE) tool, including scoring values, root-mean-square distance, and (RMSD) values, are given in Table 1. As reported earlier [35], the more negative the free binding affinity/scoring values are, the better the bond stability it forms between the ligand and the receptor protein [35]. In this context, strong relations between POPs and ERα protein of breast cancer were assessed based on scoring values ranging from −31.94 to −8.650 kcal/mol. Out of 27 POPs, short-chained chlorinated paraffins (SCCPs), hexabromocyclododecane (HBCD), and perfluorooctanesulfonyl fluoride (PFOSF) showed the strongest molecular interactions with the ERα protein based on their lowest-scoring values of −31.94, −18.91, −17.58 kcal/mol, respectively (Table 1). These findings revealed that SCCPs, HBCD, and PFOSF could be potentially involved in breast cancer prevailing compared to PCBs and organochlorine as reported in previous studies [34]. These POPs are more suspected to cause breast cancer and are widely used pesticides in developing countries of Asia due to their low cost and utility against various pests. The key non-occupational exposure routes of these high-potency contaminants include ingestion, both directly and by tainted food, and dermal interaction with the substance [36].

**Figure 1:**
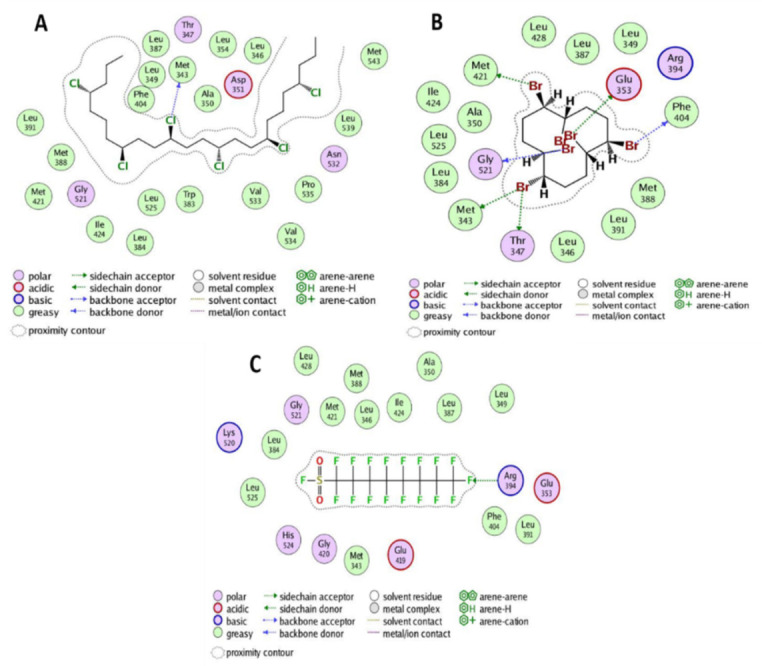
Two dimensional interactions of short chain chlorinated paraffins (**A**), Hexabromocyclododecane (**B**) and Perfluorooctanesulfonyl fluoride (**C**) with ERα protein of Breast Cancer.

Above mentioned results revealed that based on scoring values, short-chain chlorinated paraffin (SCCPs) have the strongest interactions with the ERα protein (Figure 1A). SCCPs are commonly used in metalworking fluids, paints, sealants, adhesives, leather manufacturing chemicals, plastics, rubber, and as a plastic agent and flame retardant [37]. In addition, these pollutants have also been isolated from kidneys, adipose tissue, and breast milk of Inuit women [38]. As previously reported, SCCPs (C10–C13) belong to the third class of carcinogens and are highly toxic to aquatic organisms [39]. Moreover, previous reports have provided convincing evidence on the disruptive effect of SCCPs on thyroid hormones and glucocorticoids [40], as well as their role in the regulation of different signaling pathways and physiological mechanisms [41]. However, there are few studies on the health effects of SCCPs in humans, especially their contribution to endocrine disruption. In this context, the aforementioned results revealed the strong association of SCCPs with the ERα protein of breast cancer based on least binding energy or scoring value (−31.946 kcal/mol). This indicates that the strong binding capacity of SCCPs with ERα protein may be involved in the spread of breast cancer in women. Hexabromocyclododecane (HBCD) has also demonstrated strong interactions with the ERα protein (Figure 1B). Therefore, HBCD could also be one of the main contributors to breast cancer.

These POPs are extensively used in flame retardants, as a neurotoxin, and in xenobiotic chemicals. HBCD acts as a nuclear receptor agonist, is hepatotoxic, and is an endocrine disruptor that induces developmental neurotoxicity in animals [42]. HBCD concentration in human breast milk, blood serum, and the umbilical cord has already been investigated. The concentrations in human breast milk raise questions about possible lactation and prenatal uptake during important developmental stages of the fetus [43]. Likewise, several fluorinated compounds, specifically perfluorooctanesulfonyl fluoride (PFOSF), also showed strong interactions with the ERα protein, based on a low scoring value (−17.58 kcal/mol), which allows it to easily form a stable complex (Figure 1C). These compounds are used as impregnating and grading agents and corrosion inhibitors, like insecticides and flame retardants, in cosmetics, paper coatings, and surfactants. The possible association of PFOSF with hormone disorders, genotoxic potential, and tumor formation in rodents has been suggested previously [44]. Hormones can be indirectly imitated by endocrine disorders or hormone disorders. Strong associations of SCCPs, HBCD, and PFOSF with the ERα protein revealed their possible role in breast cancer; therefore the role of these POPs should be further investigated in detail.

### 3.3. Natural and Synthetic Compounds Collection, Mining, and Clustering

Approximately 7856 natural and synthetic compounds were collected with their structures and properties using the ZINC database (zinc.docking.org/ (accessed on 12 July 2020)). While applying the Lipinski rule of five on the drug dataset, 2390 compounds were chosen for further processing and the rest were discarded after mining. Afterward, 12 clusters were generated for natural and synthetic compounds based on the k-means clustering system using the Weka tool. The use of k-means clustering avoids the repetition of compounds, so natural and synthetic compounds exhibiting similar properties were placed in one group. Clusters possessing similar properties, including molecular weight, hydrogen bond donors log P, polar dissociation, rotatable bonds, a-polar dissociation, and hydrogen bond acceptors (HBA) are shown in a plot matrix with their attributes utilizing different color representations (Figure S3).

### 3.4. DDI Network

**Figure 2:**
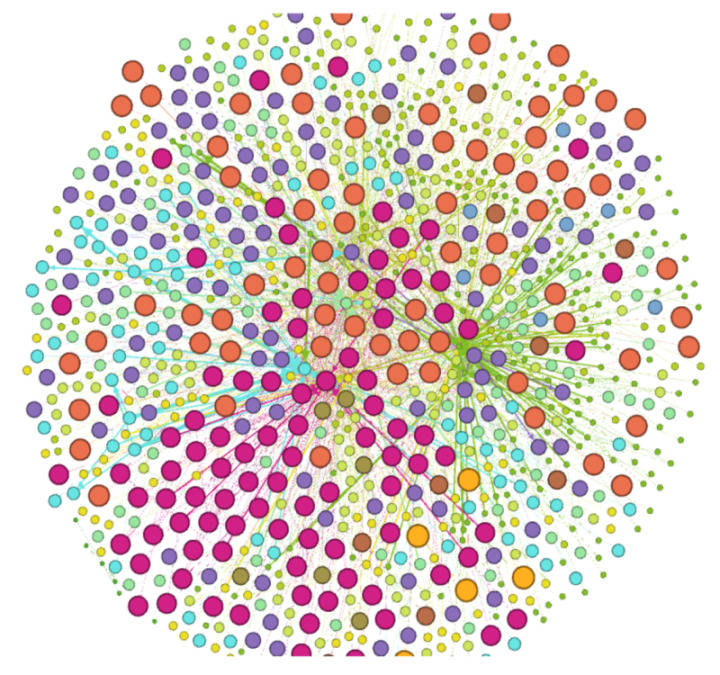
Final strongly drug-drug interaction network.

In total, 12 networks were generated using the k-means clustering algorithm and Gephi tool (Figure S4) based on statistical parameters as mentioned in Table 2. A strongly interacted network as illustrated in Figure 2 was generated from 457 compounds having higher modularity class in 12 networks based on the aforementioned parameters. Repulsion strength was set to 10,000, and Force Atlas and Fruchterman rein gold layouts were used to display and visualize the networks.

Nodes and edges in each network represent compounds and interactions between them, respectively. Darker color and larger size nodes show the strength of drug IDs/ZINC IDs. As shown in Table 2, network 1 comprises of 507.0 nodes and 1169 edges with modularity of 0.518; the second network consists of 266 nodes and 564 edges with 0.524 modularity. Network 3 showed 185.0 nodes and 383.0 edges with 0.538 modularity. Network 4 comprises 64.00 nodes and 98.00 edges with 0.613 modularity. Network 5 contains 923.0 nodes and 2146 edges with a modularity of 0.509. Network 6 consists of 188 nodes and 395.0 edges with a modularity of 0.571. Network 7 comprises 469.0 nodes and 1067 edges and possesses a modularity of 0.525. Network 8 consists of 107.0 nodes and 181.0 edges, and 0.001 graph density with a modularity of 0.552. Network 9 comprises 369.0 nodes and 870.0 edges with modularity of 0.500. Network 10 consists of 541.0 nodes and 1166 edges with a 0.501 modularity. Network 11 comprises 218.0 nodes and 331.0 edges and 0.528 modularity, while network 12 contains 300.0 nodes, 666.0 edges, and 0.500 modularity. Likewise, the finally generated strong network contains 923.0 nodes and 2166 edges with 0.503 modularity (Table 2).

### 3.5. Validation of Natural and Synthetic Compounds

**Figure 3:**
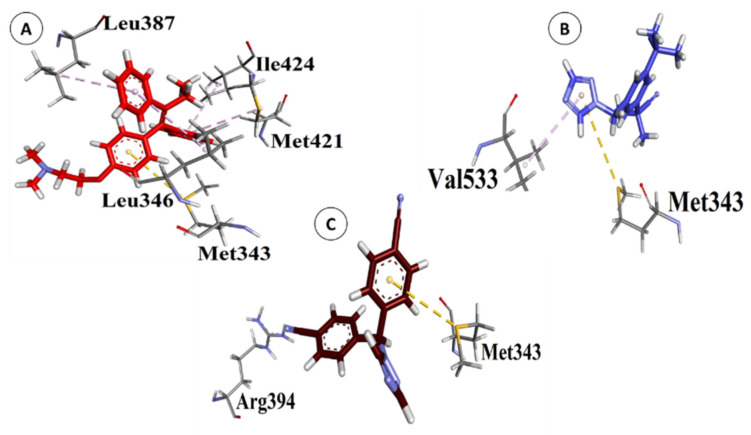
Three-dimensional interactions of Tamoxifen (**A**), Arimidex (**B**), Letrozole (**C**) with the binding site of ERα protein.

ERα, the most common and effective target for breast cancer treatment, was docked against the screened natural and synthetic compounds [35]. For docking, the active site of the ERα protein was identified using standard drugs used to treat breast cancer i.e., Tamoxifen, Arimidex, and Letrozole as a positive control group, while progesterone and testosterone were utilized as a negative control group. As shown in Table 3, scoring values of Tamoxifen, Arimidex and Letrozole ranged from −31.26 to −20.97 kcal/mol. Tamoxifen showed pi-sulfur and pi-alkyl interactions; Arimidex also showed pi-sulfur and pi-alkyl interactions, while Letrozole showed conventional hydrogen bond and pi-sulfur interactions with the active site of ERα, as shown in Figure 3a–c.

Progesterone and testosterone were used as a negative control group as their scoring values are −23.53 and −23.95 respectively, as shown in Table 3. Previous research reveals that adding an androgen to estrogen treatment lowers mammary epithelial proliferation and ER expression, implying that androgens may protect against breast cancer in the same way as progesterone does. In the past, androgens were used to treat breast cancer with moderate effectiveness [45]. There are mixed theories about these hormones, as some claim they down-regulate breast cancer progression, whereas some claims they up-regulate the process. However, according to our studies, both progesterone and testosterone have shown interactions with ER alpha protein. Furthermore, this study reveals that these hormones may up-regulate breast cancer development, as they interacted at the active site of ER-alpha with binding energies of −23.53 (progesterone) and −23.95 (testosterone). Up-regulation of breast cancer due to an imbalance of these hormones has already been reported. It has been reported that the effects of sex hormones on breast cancer development are clear from the advantages of hormone withdrawal treatment, with particular evidence of a relationship between completion of hormone withdrawal and clinical benefit [46].

The majority of clinical investigations that have utilized total testosterone as a measure of androgen exposure have found that greater total testosterone levels are related to an increased risk of breast cancer [47]. Progesterone metabolites may consequently be involved in the regulation of the generation of estradiol in the normal breast cell and so may be a multifaceted component in breast carcinogenesis [48]. Progesterone triggers normal human breast epithelial through paracrine mechanisms and is a risk factor for breast cancer because it promotes pre-neoplastic progression by stimulating cyclic proliferation of mammary stem cell pools or cell-initiating tumors in maturing breast epithelium. The development of cancer is therefore further promoted by progesterone signaling and a transition to autocrine proliferation regulation [49].

Natural and synthetic compounds possessing the highest binding affinity, RMSD less than 2 Å, and accurate binding sites were considered to form the most stable complexes. Data of 61 natural and synthetic compounds were collected from strong DDI networks and the top five synthetic (ZINC08441573, ZINC00664754, ZINC00702695, ZINC00627464, ZINC08440501) and four nature compounds (capsaicin, flavopiridol, tectorigenin, and ellagic acid) were docked with ERα (Table 3). Based on their scoring values and RMCD, both synthetic and natural compounds depicted strong binding capacity with the active site of the ERα protein.

**Figure 4:**
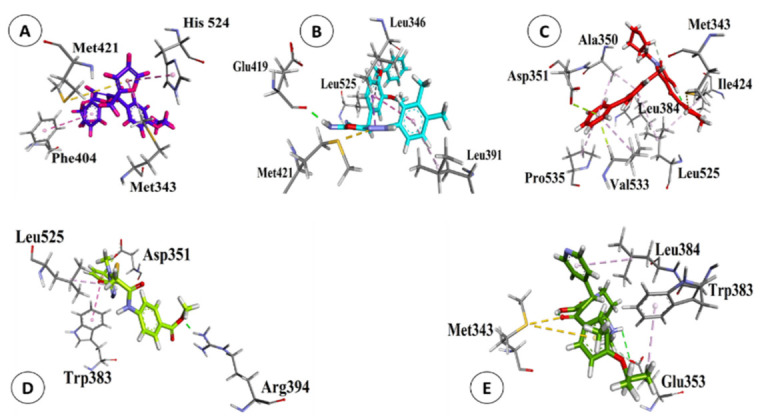
Three-dimensional interactions of synthetic compounds ZINC08441573 (**A**), ZINC00664754 (**B**), ZINC00702695 (**C**), ZINC00627464 (**D**), and ZINC08440501 (**E**) with the binding site of ERα protein.

Predicted scoring values of the top five synthetic compounds, ZINC08441573, ZINC00664754, ZINC00702695, ZINC00627464, and ZINC08440501, were −32.47, −31.38, −30.35, −30.31, and −29.350 kcal/mol, respectively. These findings were further confirmed by 3-D interactions with the active site of the ERα protein as shown in Figure 4a–e. The ZINC08441573 drug compound showed pi-pi, T shaped and pi-sigma interactions; ZINC00664754 exhibited conventional hydrogen bonding, pi-sigma, pi-sulphur and pi-alkyl bonding; ZINC00702695 had pi-sulfur, pi-lone pair and pi-alkyl associations; ZINC00627464 showed conventional hydrogen bonding, pi-pi T shaped, pi-lone pair and pi-alkyl associations; and ZINC08440501 had conventional hydrogen bond, Sulfur-X, pi-sulfur, and pi-alkyl interactions with the active sites of the ERα protein. Our findings suggest that, relatively, most of the synthetic compounds have scoring values even less than the standard breast cancer drugs, therefore showing strong interactions with the ERα protein. These compounds could be potential candidates to treat breast cancer. The inhibition potential of all synthetic compounds was greater than two standard breast cancer drugs i.e., Arimidex and letrozole. Two synthetic compounds including ZINC08441573 and ZINC00664754 exhibited a highly significant strong binding capacity (based on lowest scoring value) with the ERα protein. These two compounds possess the strong potential to inhibit interactions of all types of POPs (Table 1) with ERα protein and could be the best alternative to standard breast cancer drugs used currently.

**Figure 5:**
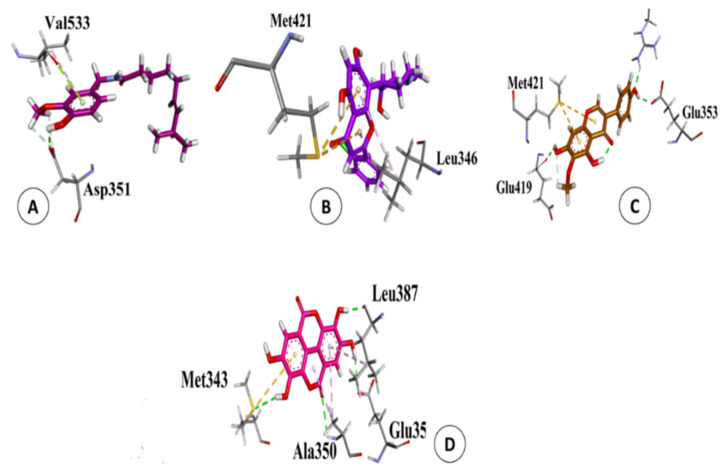
Three-dimensional interactions of natural compounds capsaicin (**A**), flavopiridol (**B**), tectorigenin (**C**), ellagic acid (**D**) with binding site of ERα protein.

Likewise, the binding capacity of the top four natural compounds from our dataset, namely capsaicin, flavopiridol, tectorigenin, and ellagic acid, with the ERα protein is demonstrated in Table 3. The scoring value of these compounds ranged from −25.30 to −16.36 kcal/mol, while their RMSD was between 0.905–1.875 Å. The association between these natural compounds and the ERα protein was further confirmed by 3-D networks as mentioned in Figure 5a–d. Capsaicin showed conventional hydrogen bonds and pi-lone pair interactions with ERα protein; flavopiridol had pi-sulfur, pi-sigma, and pi-alkylinteractions; tectorigenin exhibited conventional hydrogen bonding and pi-sulfur interactions; and ellagic acid showed conventional hydrogen bonding, pi-sulfur, and pi-alkyl interactions. Based on scoring values, it can be predicted that capsaicin and flavopiridol have a strong association with ERα protein, which is even stronger than standard drugs (Arimidex and letrozole). These natural compounds also have the potential to inhibit the binding of almost all POPs with ERα protein except short-chained chlorinated paraffins (Table 1). Based on scoring values and RMSD, the majority of the synthetic and natural compounds have the potential to inhibit various interactions between POPs and active sites of the ERα breast cancer protein. Such compounds could also be potential candidates for breast cancer drugs, and can also be useful as an alternative to standard drugs to treat breast cancer caused by POPs and other organic toxins.

## 4. Conclusions

The bioaccumulation of POPs in adipose tissue and their role in breast cancer development and/or progression was evaluated using the molecular interaction approach. Based on scoring values, short-chained chlorinated paraffins demonstrated strong interactions with the ERα breast cancer protein compared to organo-chlorines and PCBs. Both synthetic and natural compounds which demonstrated strong associations with the active site of the ERα protein could be potential candidates to treat breast cancer specifically caused by POPs and other organic toxins, and can be used as an alternative to standard drugs. Furthermore, our findings could be validated using in vitro and in vivo approaches.

## Figures and Tables

**Table 1:** Docking of POPs with ERα protein.

S. #	Chemical Name	Structures	B.E. (kcal/mol)	RMSD (Å)
1.	Short-chained chlorinated paraffin’s	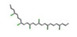	−31.95	1.975
2.	HBCD (Hexabromocyclododecane)	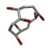	−18.92	0.831
3.	PFOSF (Perfluorooctanesulfonyl fluoride)	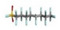	−17.58	1.920
4.	Dieldrin	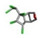	−17.22	0.635
5.	DDT (dichloro-diphenyl-trichloroethane)	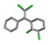	−17.15	1.123
6.	PFOS (perfluorooctanesulfonic acid)	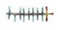	−17.13	0.884
7.	Endrin	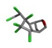	−17.01	0.979
8.	Aldrin	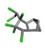	−16.19	1.312
9.	Hexa bromodiphenyl ethers	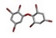	−15.72	1.099
10.	Hexabromobiphenyl	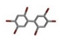	−15.67	1.541
11.	Penta-bromodiphenyl ethers	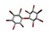	−15.39	1.261
12.	PCDDs (Polychlorinated dibenzodioxins)	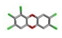	−15.01	1.345
13.	Chlordane	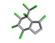	−14.85	0.937
14.	Toxaphene	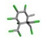	−14.55	0.818
15.	PCDFs (polychlorinated dibenzofurans)	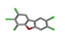	−14.39	1.885
16.	Beta endosulfans	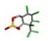	−14.26	1.791
17.	PCBs (Polychlorinated biphenyls)	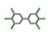	−14.16	1.233
18.	α-endosulfans	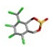	−14.09	0.978
19.	Heptachlor	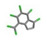	−13.70	0.999
20.	Lindane	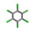	−12.91	0.631
21.	Chlorinated_naphthalenes	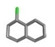	−12.58	0.903
22.	Mirex	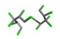	−12.39	1.508
23.	Chlordecone	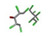	−11.87	1.207
24.	Pentachlorophenol	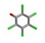	−9.566	1.215
25.	Hexachlorobenzene	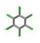	−9.501	1.212
26.	Pentachlorobenzen	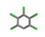	−9.500	1.596
27.	Hexachlorobutadiene	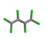	−8.650	1.090

B.E. Binding energy, RMSD. Root-mean-square distance, S. #. Serial number.

**Table 2:** Statistical parameters used to predict network interactions of various natural and synthetic compounds.

Networks	AD	AWD	ND	GD	Ml	APL	N	E
1.	2.306	2.860	1.000	0.005	0.518	1.000	507.0	1169
2.	2.120	2.519	1.000	0.008	0.524	1.000	266.0	564.0
3.	2.070	2.649	1.000	0.011	0.538	1.000	185.0	383.0
4.	1.531	2.266	1.000	0.024	0.613	1.000	64.00	98.00
5.	2.325	2.476	1.000	0.003	0.509	1.000	923.0	2146
6.	2.101	3.005	1.000	0.011	0.571	1.000	188.0	395.0
7.	2.275	3.028	1.000	0.005	0.525	1.000	469.0	1067
8.	1.629	2.056	1.000	0.001	0.552	1.000	107.0	181.0
9.	2.371	2.792	1.000	0.006	0.500	1.000	367.0	870.0
10.	2.155	2.662	1.000	0.004	0.501	1.000	541.0	1166
11.	1.518	1.789	1.000	0.007	0.528	1.000	218.0	331.0
12.	2.220	2.967	1.000	0.007	0.500	1.000	300.0	666.0
FSIN	2.325	2.476	1.000	0.003	0.503	1.000	923.0	2146

AD. Average degree, AWD. Average weighted degree, ND. Network diameter, GD. Graph density, Ml. Modularity, APL. Average path length, N. Nodes, E. Edges, FSIN. Final strongly interacted network.

**Table 3:** Docking results of positive control, negative control, synthetic and natural compounds with ERα protein.

S. #	Names	Structures	B.E. (kcal/mol)	RMSD (Å)
Positive control				
1	Tamoxifen	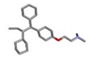	−31.26	1.006
2	Arimidex	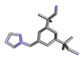	−22.46	1.130
3	Letrozole	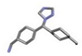	−20.97	1.385
Negative control				
1	Progestrone	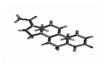	−23.53	1.080
2	Testosterone	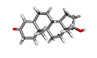	−23.95	0.859
Synthetic compounds				
1	5-(4-butoxyphenyl)-4-(2,3-dihydro-1,4-benzodioxin-6-ylcarbonyl)-1-(2-furylmethyl)-3-hydroxy-1,5-dihydro-2*H*-pyrrol-2-one [ZINC08441573]	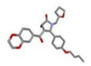	−32.47	1.521
2	2-(4-*tert*-butylphenyl)-3*H*-quinazolin-4-one [ZINC00664754]	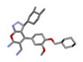	−31.38	1.575
3	(2*S*)-4-hydroxy-3-(5-methylfuran-2-carbonyl)-1-[[(2*R*)-oxolan-2-yl]methyl]-2-(3-phenoxyphenyl)-2*H*-pyrrol-5-one [ZINC00702695]	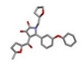	−30.35	1.820
4	(1*R*,6*S*)-6-[[2-[4-(4-methylphenyl) piperazine-1-carbonyl]phenyl]carbamoyl]cyclohex-3-ene-1-carboxylic acid [ZINC00627464]	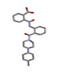	−30.31	1.650
5	(5*S*)-1-[3-(dimethylamino)propyl]-4-[hydroxy-(3-methyl-4-propan-2-yloxyphenyl)methylidene]-5-pyridin-4-ylpyrrolidine-2,3-dione [ZINC08440501]	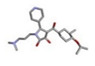	−28.76	1.686
Natural compounds				
1	Capsaicin [ZINC01530575]	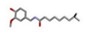	−24.90	0.905
2	Flavopiridol [ZINC21288966]	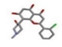	−24.76	1.875
3	Tectorigenin [ZINC00899915]	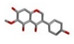	−21.71	0.994
4	Ellagic acid [ZINC03872446]	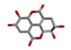	−16.36	0.996

B.E. Binding energy, RMSD. Root-mean-square distance, S. #. Serial number.

## Data Availability

The data presented in this study are available within the article and supplementary materials.

## Supplementary Materials

The following are available online at https://www.mdpi.com/article/10.3390/biology10070681/s1, Table S1: Results of re-docking of native inhibitors; Table S2: Cross-docking results for various PDB IDs from ERα; Figure S1: Structure of estrogen receptor alpha (ERα/pdb id = 5W9D) protein; Figure S2: Chemical structures and binding energies (B.E. in kcal/mol) of the active compounds obtained from CHEMBL; Figure S3. Plot matrix representation of drug compounds with their attributes; Figure S4. DDI networks generated using K means clustering algorithm and Gephi tool.
