# Supplementary material for: Role of Persistent Organic Pollutants in Breast Cancer Progression and Identification of Estrogen Receptor Alpha Inhibitors Using In-Silico Mining and Drug-Drug Interaction Network Approaches
Source: Biology (Basel). 2021 Jul 19;10(7):681. doi: 10.3390/biology10070681 (PMC8301456; doi:10.3390/biology10070681)
Supplement: Supplementary file 1 [file biology-10-00681-s001.zip › biology-1238538-supplementary.pdf]

biology MDPI

**Docking validation**

We performed docking on ER alpha. First, we performed docking validation on *ER alpha*. The reliability of docking accuracy was assessed in two steps. In the first step re-docking of the native ligand was performed. While, in the second step, cross-docking experiment was carried out. Three-dimensional structures of eight ER alpha enzymes were retrieved from PDB. For every available structure, each native ligand was docked.

**1. Self-docking on *ERα***

Validation of the docking protocol was carried out using re-dock procedure. All the native ligands were extracted and redocked into corresponding enzyme. The root means square deviation (RMSD) was calculated for the each re-docked and experimental native ligand. Triangle Matcher place algorithm with Affinity *d*G scoring function for all the simulation was found best. Final score function was computed with GBVI/WAS *d*G score function in the rigid receptor protocol.

**Table S1.** Results of re-docking of native inhibitors.

| PDB ID | RMSD (Å)* |
|---|---|
| 1A52 | 0.69 |
| 3ERT | 1.65 |
| 1GWQ | 0.98 |
| 1UOM | 1.01 |
| 5W9D | 0.97 |

* Green box = Good pose; Yellow box = close pose.

**2. Cross docking on *h*DHFR**

Cross-docking experiment was performed in the next step. Three-dimensional structures of five ER*α* were retrieved from PDB. For every available structure, each native ligand was docked. The quality of docking accuracy / docking pose was assessed with the following RMSD values range.

≤ 1.10 = Good pose (Green box)
< 1.11-1.90 = close pose (yellow box)
≥ 2.00 bad pose (Red box)

**Table S2.** Cross-docking results for various PDB IDs from ER*α*.

| | RMSD (Å) | | | | |
|---|---|---|---|---|---|
| | **1A52** | **3ERT** | **1GWQ** | **1UOM** | **5W9D** |
| **1A52** | 0.69 | 0.96 | 0.98 | 0.82 | 0.99 |
| **3ERT** | 1.18 | 0.91 | 1.48 | 1.28 | 1.01 |
| **1GWQ** | 1.18 | 1.57 | 1.36 | 0.78 | 1.36 |
| **1UOM** | 1.18 | 1.97 | 1.82 | 1.06 | 2.13 |
| **5W9D** | 1.09 | 1.15 | 1.04 | 0.84 | 1.04 |

≤ 1.10 = Good pose (Green box); < 1.11-1.90 = close pose (yellow box); ≥ 2.00 bad pose (Red box).

The results shown in **Table S2** indicates that docking simulations carried out on 3-D structures in complex with different ligand had only about 64 % of chance of reliable pose. It is revealed from the cross-docking experiment that for 1A52 and 5W9D, it is possible to dock five ligands with RMSD range of 0.81-1.37 Å. Furthermore, the native ligand of 1DLS is MTX that has structural similarity with our synthesized ligands.

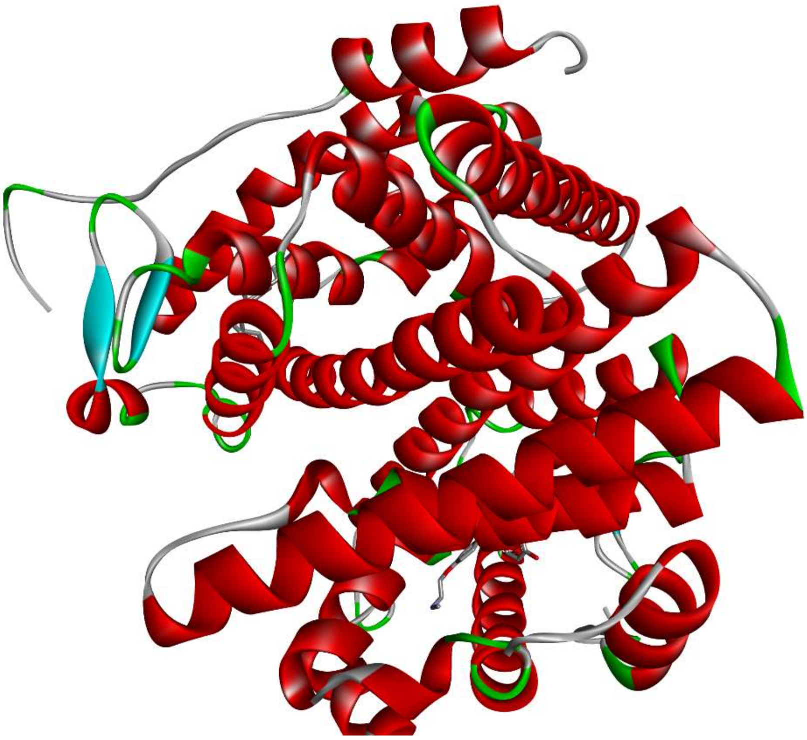

**Figure S1.** Structure of estrogen receptor alpha (ER$\alpha$/pdb id=5W9D) protein.

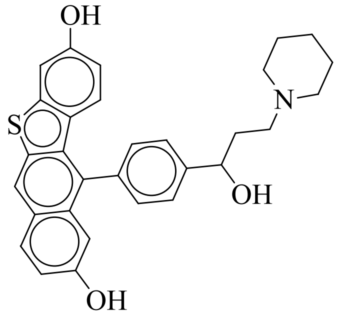

**CHEMBL-182342**
**B.E. = -30.8013**

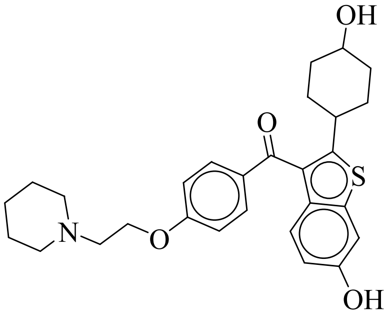

**CHEMBL-64692**
**B.E. = -33.0997**

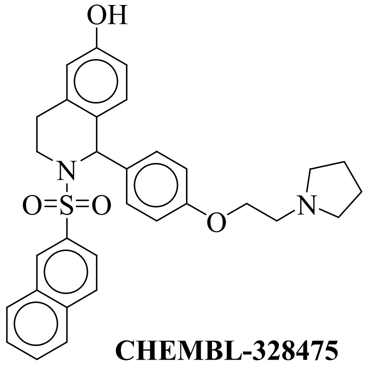

**CHEMBL-328475**
**B.E. = -33.4849**

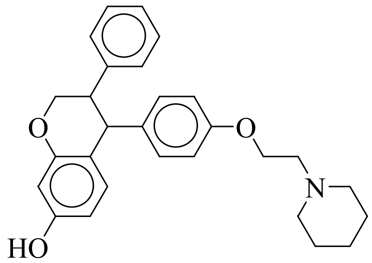

**CHEMBL-299942**
**B.E. = -32.1575**

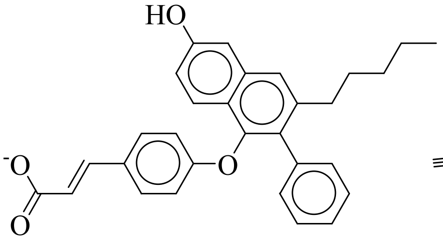

**CHEMBL-481189**
**B.E. = -35.9801**

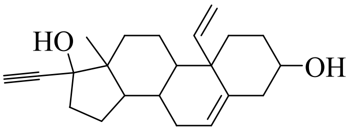

**CHEMBL-381566**
**B.E. = -30.6157**

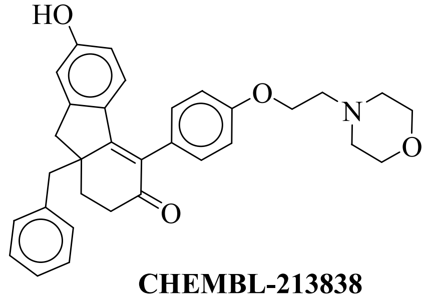

**CHEMBL-213838**
**B.E. = -34.0694**

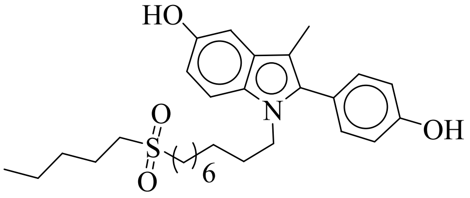

**CHEMBL-184074**
**B.E. = -30.0541**

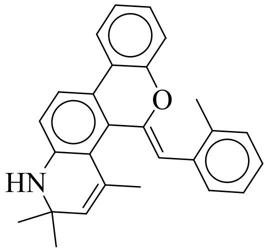

**CHEMBL-3023628**
**B.E. = -30.8631**

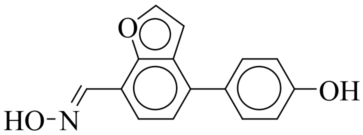

**CHEMBL-231812**
**B.E. = -30.3861**

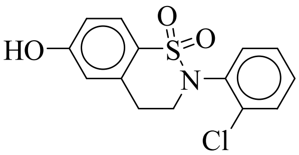

**CHEMBL-378581**
**B.E. = -30.3345**

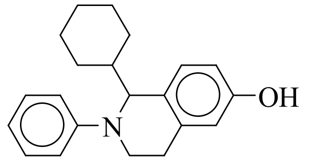

**CHEMBL-412907**
**B.E. = -30.6704**

**Figure S2.** Chemical structures and binding energies (B.E. in kcal/mol) of the active compounds obtained from CHEMBL.

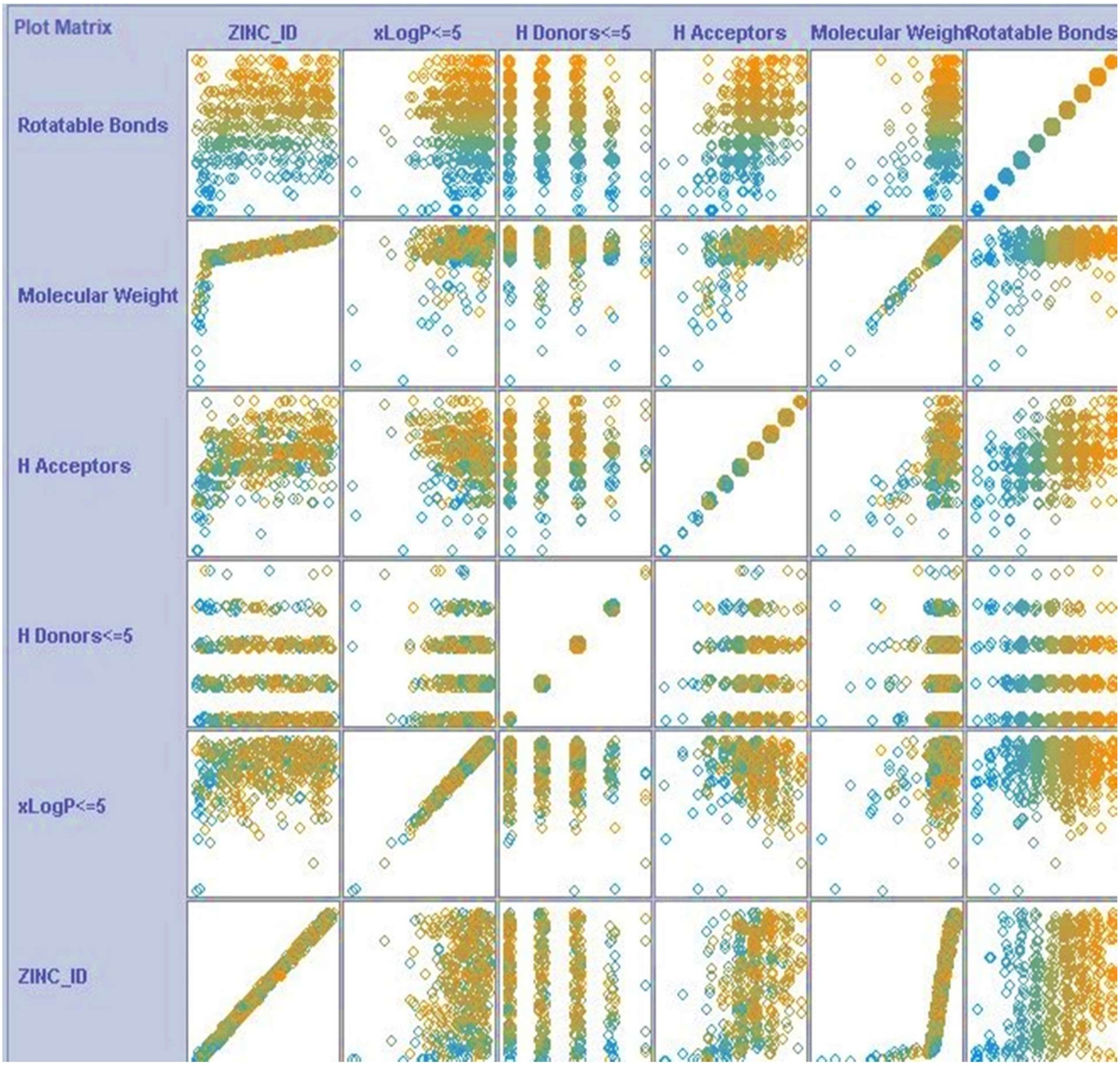


***Figure S3.*** Plot matrix representation of drug compounds with their attributes.

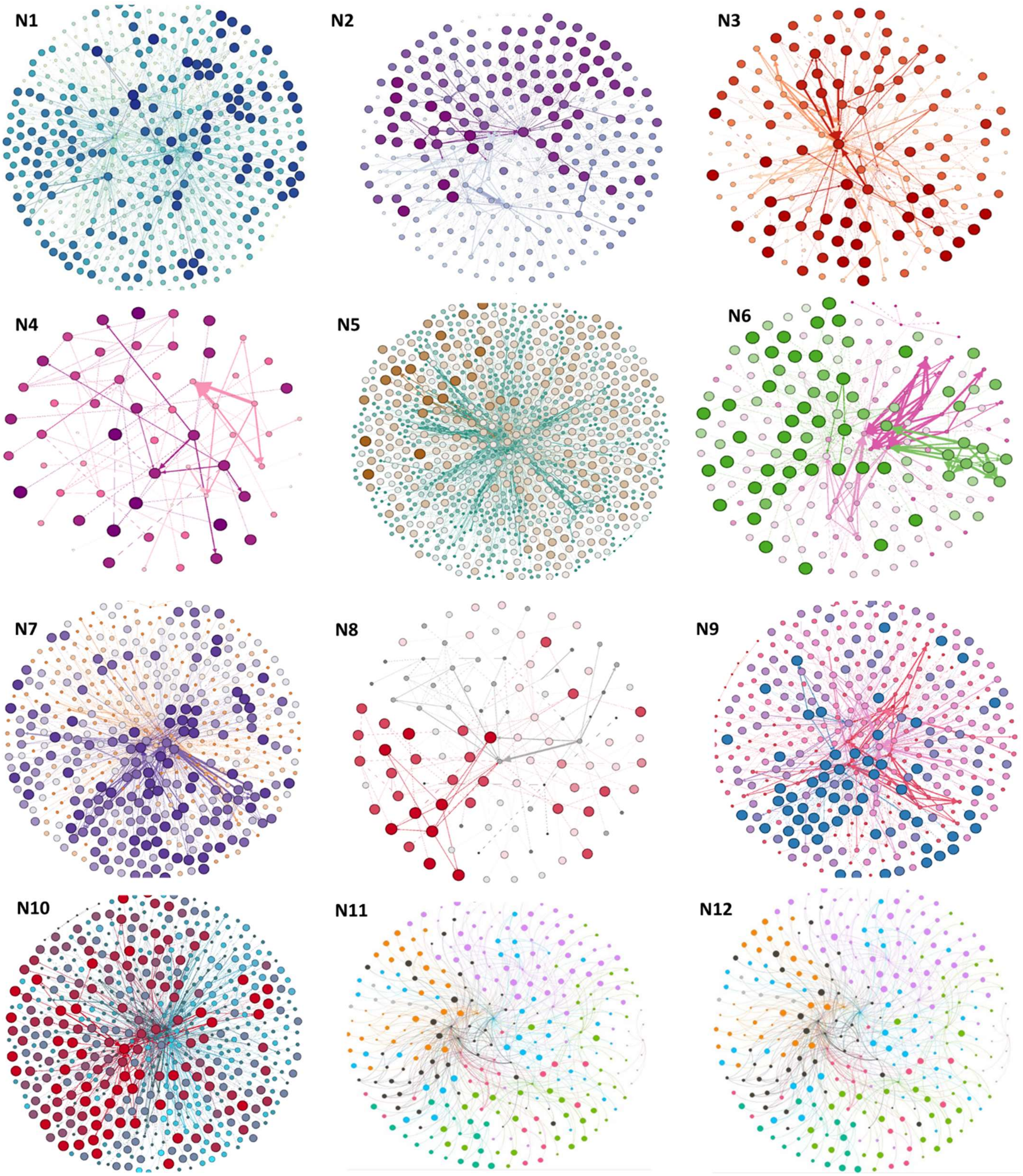


**Figure S4.** DDI networks generated using K means clustering algorithm and Gephi tool.
